# MiR-145 Inhibits Metastasis by Targeting Fascin Actin-Bundling Protein 1 in Nasopharyngeal Carcinoma

**DOI:** 10.1371/journal.pone.0122228

**Published:** 2015-03-27

**Authors:** Ying-Qin Li, Qing-Mei He, Xian-Yue Ren, Xin-Ran Tang, Ya-Fei Xu, Xin Wen, Xiao-Jing Yang, Jun Ma, Na Liu

**Affiliations:** Sun Yat-sen University Cancer Center, State Key Laboratory of Oncology in South China, Collaborative Innovation Center of Cancer Medicine, Guangzhou, People’s Republic of China; Peking University Cancer Hospital & Institute, CHINA

## Abstract

**Background:**

Based on our recent microarray analysis, we found that miR-145 was obviously downregulated in nasopharyngeal carcinoma (NPC) tissues. However, little is known about its function and mechanism involving in NPC development and progression.

**Methods:**

Quantitative RT-PCR was used to detect miR-145 expression in NPC cell lines and clinical samples. Wound healing, Transwell migration and invasion, three-dimension spheroid invasion assays, and lung metastasis model were performed to test the migratory, invasive, and metastatic ability of NPC cells. Luciferase reporter assay, quantitative RT-PCR, and Western blotting were used to verify the target of miR-145.

**Results:**

MiR-145 was obviously decreased in NPC cell lines and clinical samples (*P*<0.01). Ectopic overexpression of miR-145 significantly inhibited the migratory and invasive ability of SUNE-1 and CNE-2 cells. In addition, stably overexpressing of miR-145 in SUNE-1 cells could remarkably restrain the formation of metastatic nodes in the lungs of mice. Furthermore, fascin actin-bundling protein 1 (*FSCN1*) was verified as a target of miR-145, and silencing *FSCN1* with small RNA interfering RNA could suppress NPC cell migration and invasion.

**Conclusions:**

Our findings demonstrated that miR-145 function as a tumor suppressor in NPC development and progression via targeting *FSCN1*, which could sever as a potential novel therapeutic target for patients with NPC.

## Introduction

Nasopharyngeal carcinoma (NPC) is a relatively common malignant tumor in Southeast Asia, especially in Southern China. Radiotherapy has been the primary treatment for patients with NPC because of its inherent anatomic constraints and high degree of radiosensitivity. Recently, more and more evidence indicates that chemoradiotherapy is the optimal treatment choice for NPC patients with advanced disease stage and its five-year survival rate has been significantly improved; however, distant metastasis is still one of the most common failure patterns [1–4]. Therefore, best understanding of the molecular mechanisms that involved in NPC metastasis is essential for the development of novel therapeutic strategies.

Metastasis is the major feature of malignant tumors and the major cause of cancer-related deaths [5]. Metastasis is a multi-step process, including detachment of cancer cells from the primary sites, intravasation into and dissemination through the vasculature, and finally proliferation and formation of secondary tumors [6]. Recently, researches about genes and gene products that drive the metastasis have been performed; however, the underlying molecular mechanisms are still elusive. Increasing evidence has demonstrated that in addition to genetic alteration, miRNAs also take part in the regulation of cancer pathological processes, especially tumor metastasis [7–9]. MiRNAs are small non-coding RNAs that repress gene expression via mRNA degradation or translational inhibition based on base pairing to the 3′ untranslated region (3′ UTR) of their target mRNAs [10]. Abnormal expression of miRNAs has been reported in most types of human cancers [11–12], including NPC [13–16]. To date, several dysregulated miRNAs have been demonstrated to be involved in NPC cell migration, invasion and metastasis, such as miR-29c, miR-451, miR-10b, miR-93, and miR-124 [17–21]. These results suggest that miRNAs play important roles in NPC tumorigenesis and progression, however, the roles of miRNAs involved in NPC carcinogenesis warrant further investigations.

Based on our recent microarray analysis, we discovered that miR-145 was significantly downregulated in NPC tissues. A series of studies have reported that miR-145 is frequently decreased in many malignancies, including breast, colon, liver, prostate, and gastric cancers, and functions as a tumor suppressor by inhibiting tumor cell growth, invasion, metastasis, and tumorigenesis, regulating cell apoptosis, cell cycle, and epithelial to mesenchymal transition [22–28]. However, the effect and mechanisms of miR-145 dysregulation involved in NPC development and progression remain unknown. Therefore, in this study, we investigated the effects of miR-145 on NPC cell migration, invasion, and metastasis. Moreover, fascin actin-bundling protein 1 (*FSCN1*) was verified as a direct functional target of miR-145. Our findings demonstrated that miR-145 function as a tumor suppressor in NPC development and progression through targeting *FSCN1*, suggesting that miR-145/*FSCN1* pathway may sever as a potential novel therapeutic target for patients with NPC.

## Materials and Methods

### Cell culture

Six human NPC cell lines (CNE-1, CNE-2, C666-1, SUNE-1, HNE-1, and HONE-1) were maintained in RPMI-1640 (Invitrogen) supplemented with 10% FBS, the immortalized human nasopharyngeal epithelial cell line NP69 was maintained in Keratinocyte/serum-free medium (Invitrogen) supplemented with bovine pituitary extract (BD Biosciences), and 293FT cell line was grown in DMEM (Invitrogen) supplemented with 10% FBS.

### Clinical samples

Thirty-six freshly-frozen biopsy NPC and fourteen normal nasopharyngeal epithelium tissue samples were collected from the pathology archives of Sun Yat-sen University Cancer Center. The protocol of this study was approved by the Institutional Ethical Review Committee of Sun Yat-sen University Cancer Center (L201402052), and written informed consent was obtained from each patient for the use of their tissue samples.

### RNA isolation and quantitative RT-PCR

Total RNA was isolated from NPC cell lines and clinical samples using TRIzol reagent (Invitrogen). 2 μg of total RNA was reverse-transcribed using M-MLV reverse transcriptase (Promega) and Bulge- Loop specific RT-primers (RiboBio) or random primers (Promega) for detecting miRNA or mRNA expression, respectively. Quantitative PCR reactions were conducted on the Bio-Rad CFX96 sequence detection system (Bio-Rad Laboratories Inc.) with Platinum SYBR Green qPCR SuperMix-UDG reagents (Invitrogen). All reactions were done in triplicate, and reactions with no template or no reverse transcriptase were used as the negative controls. The *U6* or *GAPDH* amplifications were used as endogenous controls, and the relative expression was calculated with the 2^-ΔΔCT^ equation.

### Oligonucleotide transfection

The miR-145 mimics, miR-145 inhibitor, small interfering RNA for *FSCN1* (siFSCN1), and their respective controls were purchased from GenePharma. Cell were seeded into 6-well plates the day before transfection, and then transfected with miRNA mimics (50 nM), inhibitor (100 nM), or siRNA (100 nM) using Lipofactamine 2000 reagent (Invitrogen) according to the manufacture’s protocol. The cells were harvested for assays at 48h after transfection.

### Generation of stably transfected cell lines

The sequence of pri-miR-145 was synthesized from human genomic DNA using PCR, and cloned into retroviral vector pMSCV-puromycin with *Bgl* II and *EcoR* I (New England Biolab). A plasmid mixture containing pMSCV-miR-24 or empty pMSCV vector, along with retroviral packaging vector PIK, was co-transfected into 293FT cells. After transfection, the lentiviral particles were harvested and used to infect SUNE-1 cells, and the stably transfected cells were selected with puromycin and further confirmed with quantitative RT-PCR.

### Wound healing assay

After seeded into 6-well plates, cultured until almost totally confluent, and starved for 24 h in serum-free medium, monolayer cells were scraped to generate artificial wounds with a sterile pipette tip, and the wound distances were measured at 0 and 24 h under the microscope.

### Transwell migration and invasion assay

Cells (5 × 10^4^ or 1.0 × 10^5^) were harvested and resuspended in serum-free medium, and then added into the upper chamber of Transwell chambers with polycarbonate membranes (8-μm-pore-size, Corning) coated without or with Matrigel (BD Biosciences) for migration or invasion assay after transfection. RPMI-1640 medium supplemented with 10% FBS were added into the lower chamber. After incubation for 24 h, the migrated or invaded cells were fixed, stained, and counted by averaging ten fields with an inverted microscope.

### Three-dimension spheroid invasion assay

Cells (1 × 10^4^) were harvested and resuspended in RPMI-1640 medium containing 5% FBS and 2% Matrigel (BD Biosciences), and then seed in 24-well plates coated with Matrigel. The medium was changed every other day, and representative images were capture at 2 days intervals for 1–2 weeks using an inverted microscope.

### *In vivo* lung metastasis model

Female BALB/c nude mice aged 3 to 4 weeks were purchased from Medical Experimental Animal Center of Guangdong Province (Guangzhou, China). All of the animal protocols were approved by the Institutional Animal Care and Use Committee of Sun Yat-sen University (00061378). 1 × 10^6^ SUNE-1 cells stably overexpressing miR-145 or control cells were suspended in 200 μl PBS, and then intravenously injected into nude mice through tail vein. Eight weeks later, the mice were sacrificed and their lungs were fixed, paraffin-embedded, cut, and stained with H&E staining.

### Luciferase reporter assay

The sequence of wild-type *FSCN1* 3′UTR containing putative binding sites of miR-145 was synthesized and cloned into Firely luciferase-expressing vector psiCHECK (Promega), and then mutant 3′UTR plasmid was created by site-directed mutagenesis at the four miR-145 binding sites. Subsequently, cells were transfected with the psiCHECK-FSCN1 wild-type or mutant reporter plasmid and miR-145 mimics or miRNA control, tighter with the control vector pRL-TK (Promega) using Lipofectamine 2000 reagent (Invitrogen). All experiments were done in triplicate. After 24 h, the cells were harvested and the luciferase activities were tested using the Dual-Luciferase Reporter System (Promega), according to the manufacturer's instructions.

### Western blotting

Total protein was extracted and quantified with Pierce BCA Protein Assay Kit (Thermo). Protein samples were separated with 9% polyacrylamide SDS gels and transferred to PVDF membranes (Millipore). Then, membranes were blocked with 5% fat-free milk, and incubated with rabbit monoclonal anti-FSCN1 antibody (1:5000; Epitomics) followed by secondary antibody (Sigma). The signals were determined using an enhanced chemiluminescence, and the anti-GAPDH antibody (1:5000, Abcam) was used as a loading control.

### Statistical analysis

SPSS 16.0 software was used for statistical analysis. All of the data were presented as the mean ± SD, and differences between groups were analyzed using the Student’s *t*-test. All tests were two-tailed; statistical significance was defined as *P* < 0.05.

## Results

### MiR-145 is decreased in NPC cell lines and clinical samples

**Fig 1:**
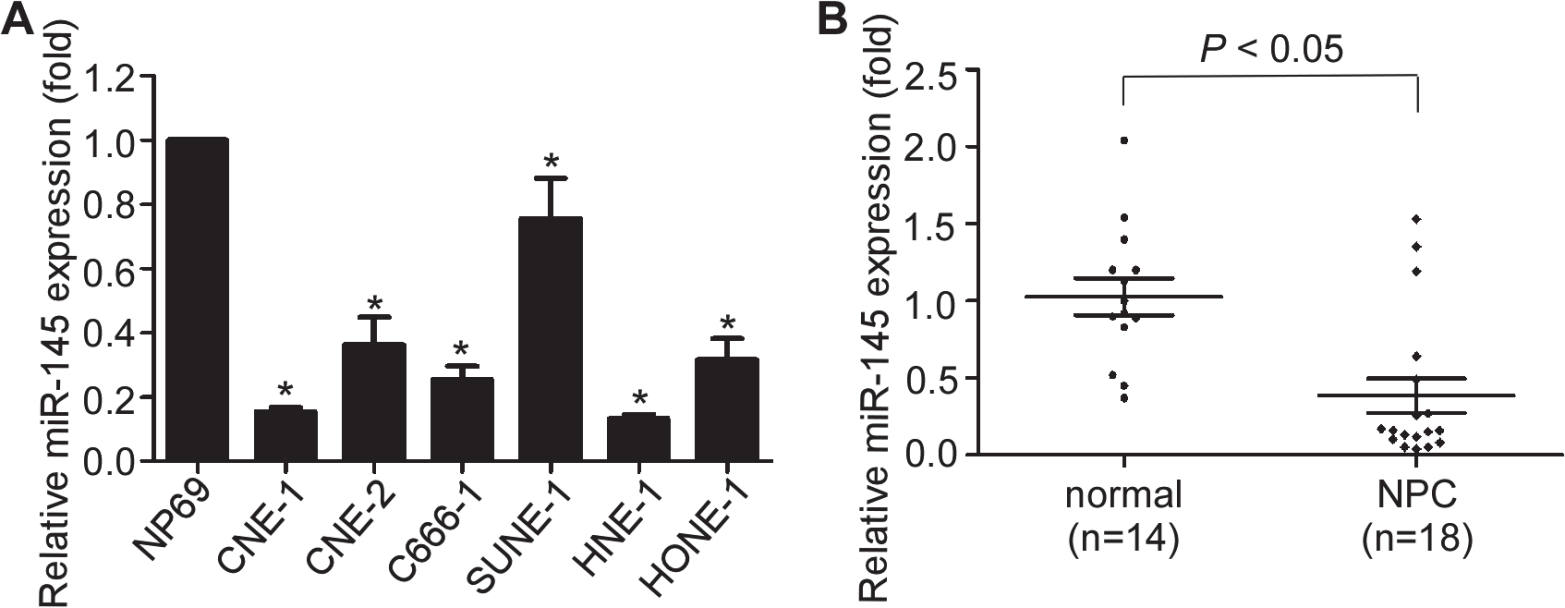
miR-145 is decreased in NPC cell lines and clinical samples. (A) Expression of miR-145 was examined in six NPC cell lines and an immortalized human nasopharyngeal epithelial cell line NP69. (B) Expression of miR-145 was tested in 18 NPC and 14 normal nasopharyngeal epithelial tissue samples (* *p* < 0.05; Student’s *t*-test).

Recently, we found that miR-145 was significantly downregulated in paraffin-embedded NPC samples based on microarray analysis. To further elucidate the significance of miR-145 in NPC, we detected the expression levels of miR-145 in a panel of human NPC cell lines and the immortalized human nasopharyngeal epithelial cell line NP69. MiR-145 was demonstrated to be remarkably decreased in all of the six NPC cell lines tested (Fig 1A). Moreover, we also determined the expression levels of miR-145 in 18 freshly-frozen biopsy NPC and 14 normal nasopharyngeal epithelial tissue samples, and found that miR-145 expression was obviously reduced in NPC tissues than in normal tissue samples (Fig 1B, *p*<0.05).

### MiR-145 suppresses NPC cell migration *in vitro*

**Fig 2:**
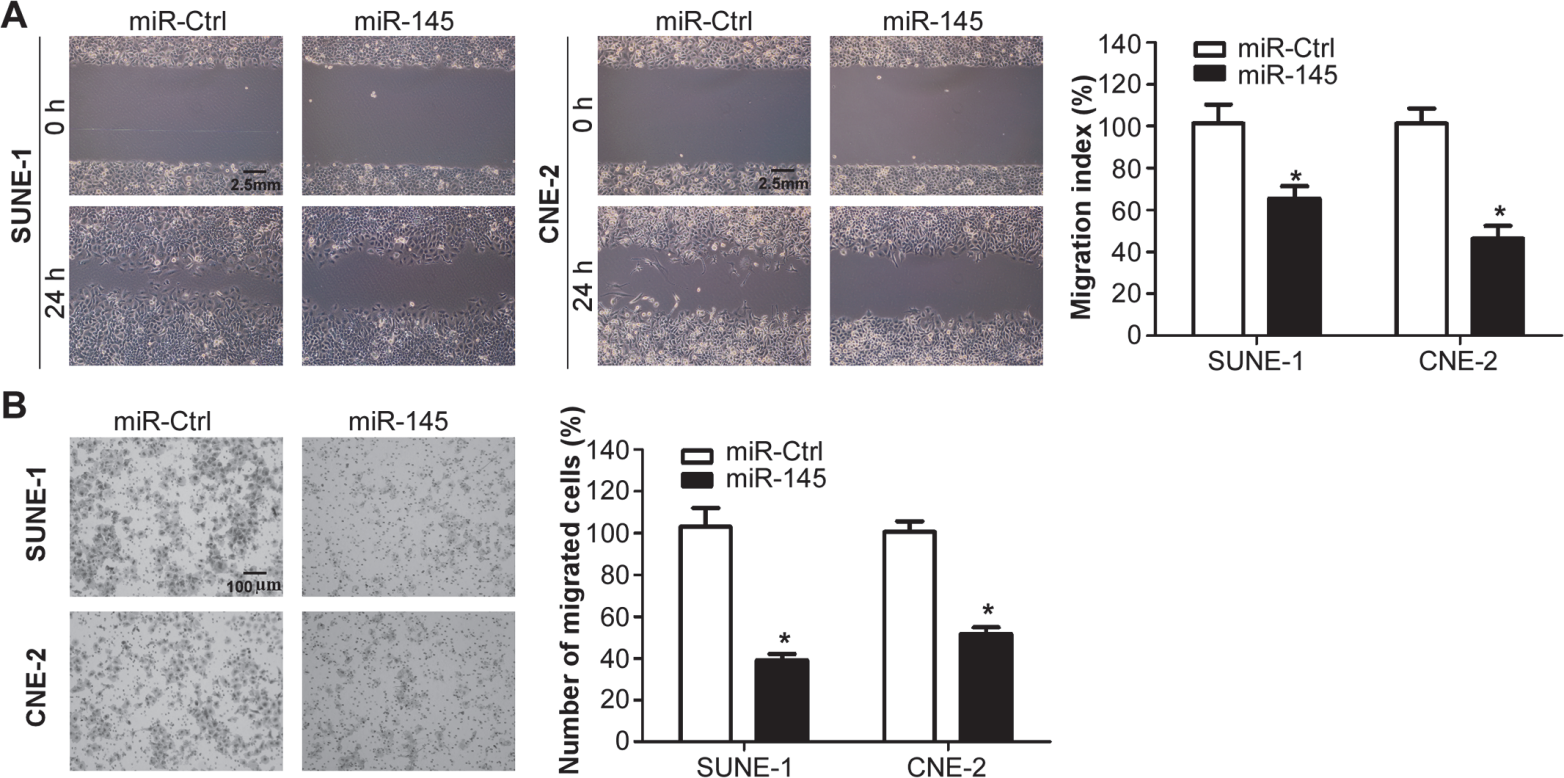
miR-145 suppresses NPC cell migration *in vitro*. (A) Representative photographs (left) and quantification (right) of the wound healing assay with SUNE-1 and CNE-2 cells transfected with miR-145 mimics or miRNA control. (B) Representative photographs (left) and quantification (right) of the Transwell migration assay with SUNE-1 and CNE-2 cells transfected with miR-145 mimics or miRNA control (* *p* < 0.05; Student’s *t*-test).

To investigate whether miR-145 affects the migratory ability of NPC cells, we transiently transfected SUNE-1 and CNE-2 cells with miR-145 mimics or miRNA control, and performed wound healing assay and Transwell migration assay. The wound healing assay demonstrated that SUNE-1 and CNE-2 cells transfected with miR-145 mimics migrated much lower than cells transfected with miRNA control (Fig 2A, both *p*<0.05). The Transwell migration assay further validated that transfection of miR-145 mimics significantly reduced the migratory ability of SUNE-1 and CNE-2 cells (Fig 2B, both *p*<0.05). These results indicated that miR-145 could significantly inhibit the migration ability of NPC cells *in vitro*.

### MiR-145 suppresses NPC cell invasion *in vitro*

**Fig 3:**
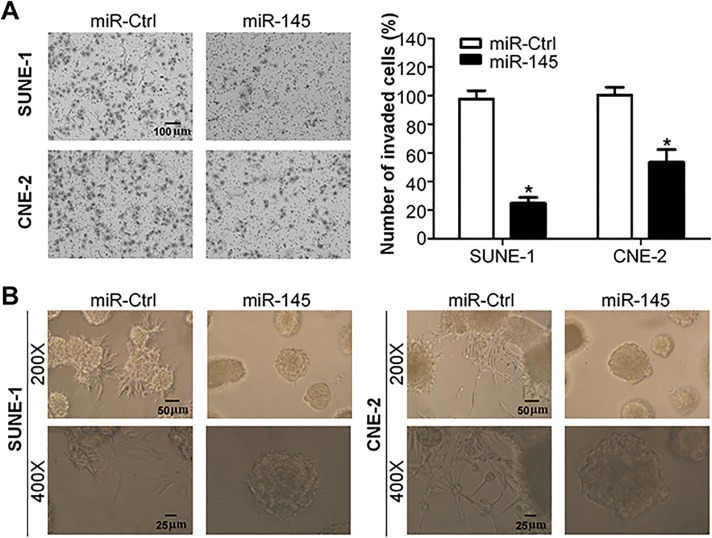
miR-145 suppresses NPC cell invasion *in vitro*. (A) Representative photographs (left) and quantification (right) of the Transwell invasion assay with SUNE-1 and CNE-2 cells transfected with miR-145 mimics or miRNA control. (B) Representative photographs of the three-dimension spheroid invasion assay with SUNE-1 and CNE-2 cells transfected with miR-145 mimics or miRNA control (* *p* < 0.05; Student’s *t*-test).

To further test whether miR-145 affects the invasive ability of NPC cells, we transiently transfected SUNE-1 and CNE-2 cells with miR-145 mimics or miRNA controls, and conducted Transwell invasion assay and three-dimension spheroid invasion assay. The Matrigel Transwell invasion assay showed that the invasive ability of NPC cells transfected with miR-145 mimics was significantly reduced than cells transfected with miRNA control (Fig 3A, both *p*<0.05). The three-dimension spheroid invasion assay indicated that overexpression of miR-145 significantly reduced the invasive ability of both NPC cells (Fig 3B). These findings suggested that miR-145 could significantly suppress the invasive ability of NPC cells *in vitro*.

### Inhibition of miR-145 promotes NPC cell migration and invasion *in vitro*

**Fig 4:**
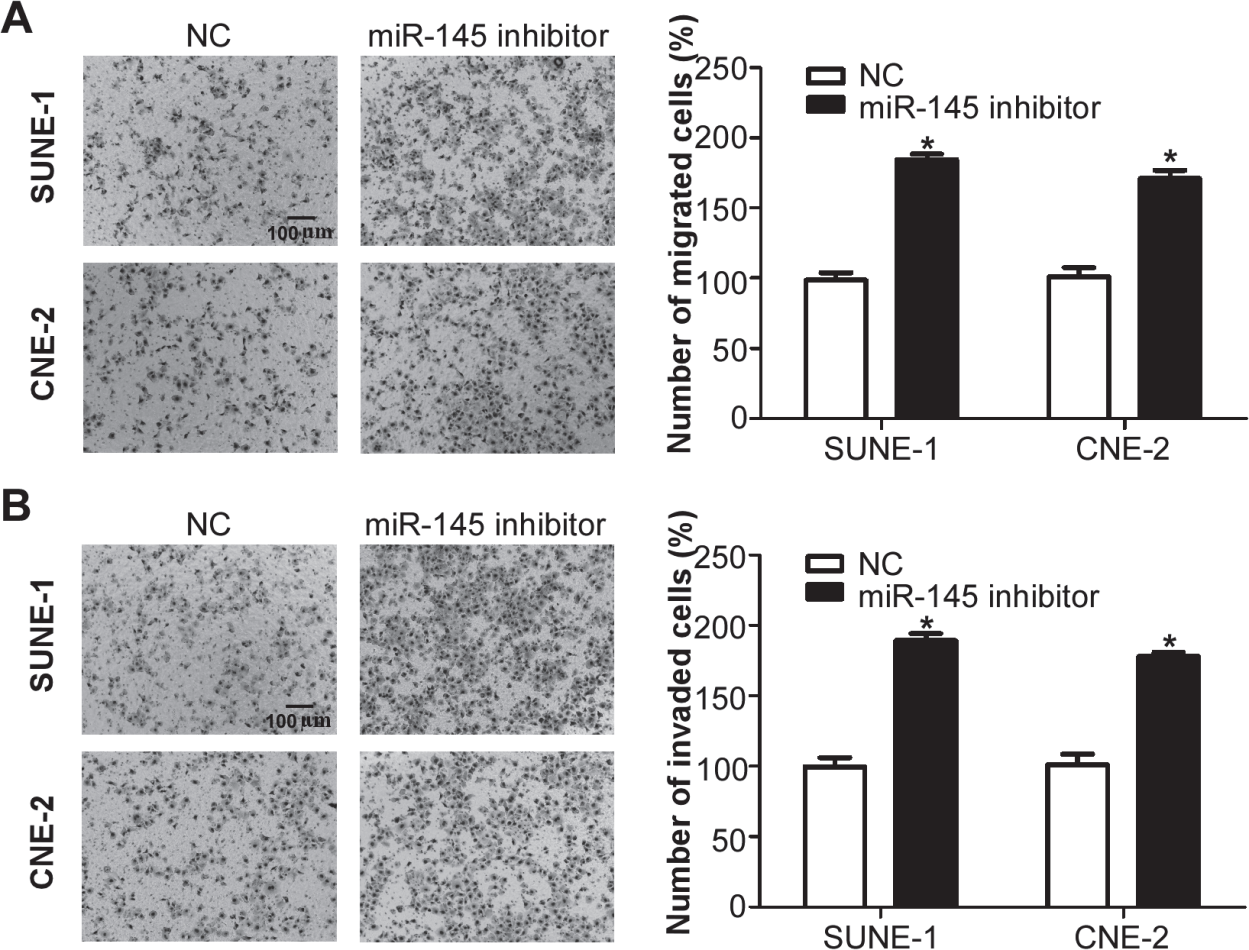
Inhibition of miR-145 promotes NPC cell migration and invasion *in vitro*. (A) Representative photographs (left) and quantification (right) of the Transwell migration assay with SUNE-1 and CNE-2 cells transfected with miR-145 inhibitor or negative control. (B) Representative photographs (left) and quantification (right) of the Transwell invasion assay with SUNE-1 and CNE-2 cells transfected with miR-145 inhibitor or negative control.

To investigate whether inhibition of miR-145 affects the migratory and invasive ability of NPC cells, we transiently transfected SUNE-1 and CNE-2 cells with miR-145 inhibitor or negative control, and performed Transwell migration and invasion assays. Transwell migration assay showed that silencing of miR-145 significantly promoted the migratory ability of SUNE-1 and CNE-2 cells (Fig 4A, both *p*<0.05). Transwell invasion assay indicated that inhibition of miR-145 also significantly promoted the invasive ability of SUNE-1 and CNE-2 cells (Fig 4B, both *p*<0.05). These results demonstrated that inhibition of miR-145 could promote the migratory and invasive ability of NPC cells.

### MiR-145 suppresses lung metastasis *in vivo*

**Fig 5:**
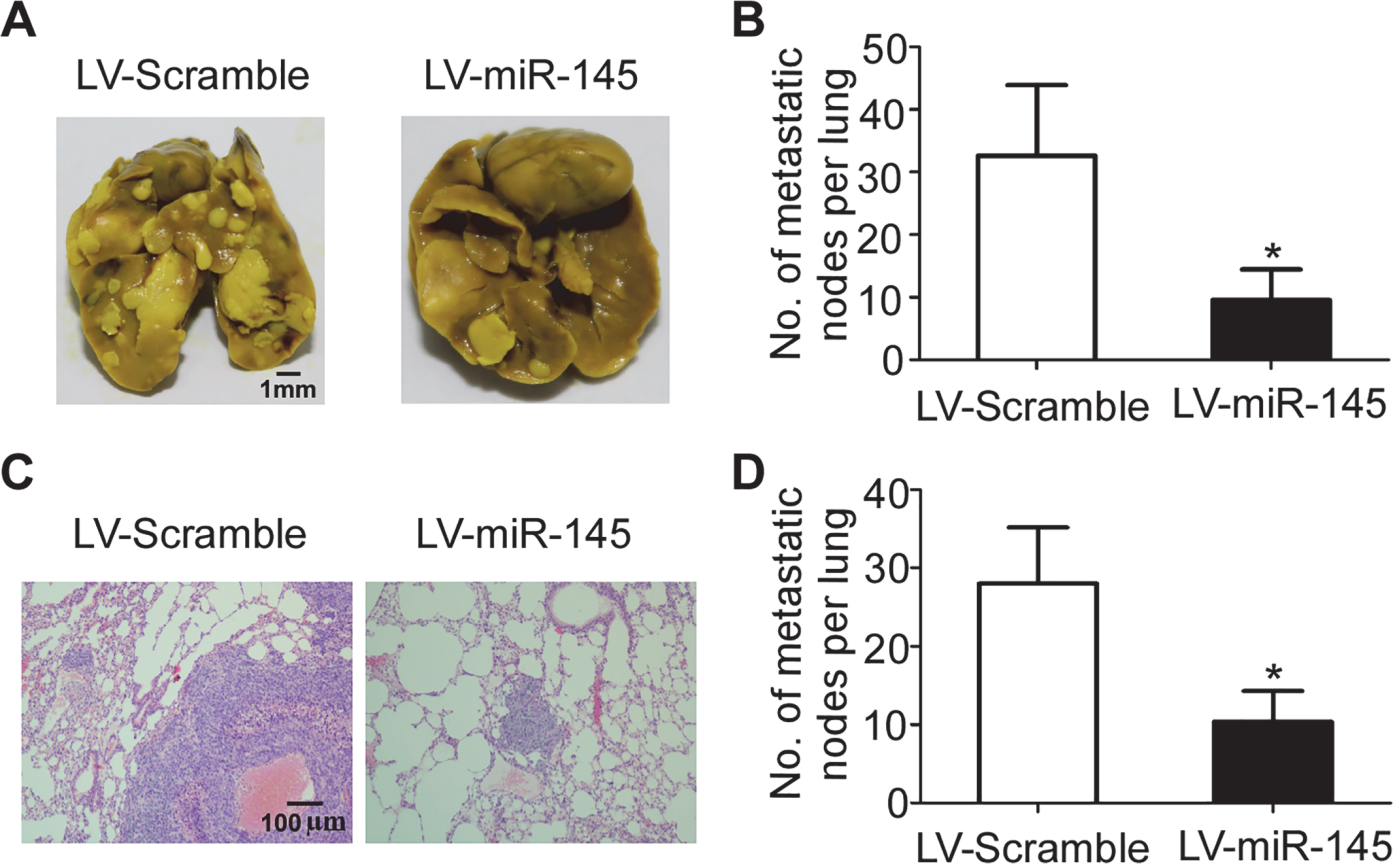
miR-145 suppresses lung metastasis in vivo. (A) Representative photographs of macroscopic metastatic nodes formed on the surface of lungs. (B) Quantification of the metastatic nodes on the surface of lungs. (C) Representative photographs of lung sections stained with hematoxylin and eosin (100×). (D) Quantification of the microscopic metastatic nodes in the lungs (* *p* < 0.05; Student’s *t*-test).

To explore the function of miR-145 in NPC metastasis in vivo, a SUNE-1 cell line stably overexpressing miR-145 was constructed using retroviral-mediated transfection, and then used to conduct an experimental metastasis assay through injecting it into the tail vein of nude mice. Eight weeks later, the mice were sacrificed and the number of metastatic nodes formed in the lungs was quantified. As shown in Fig 5A and 5B, there were fewer metastatic nodes formed on the lungs of mice injected with SUNE-1 cells stably overexpressing miR-145 (*p*<0.05). H&E staining verified that both the size and number of lung micrometastatic nodes was remarkably smaller in mice injected with SUNE-1 cells stably overexpressing miR-145 than control mice (Fig 5C and 5D, *p*<0.05). These observations demonstrated that miR-145 could obviously inhibit NPC cell metastasis, suggesting miR-145 functions as a negative modulator of metastasis.

### FSCN1 is a direct target of miR-145

**Fig 6:**
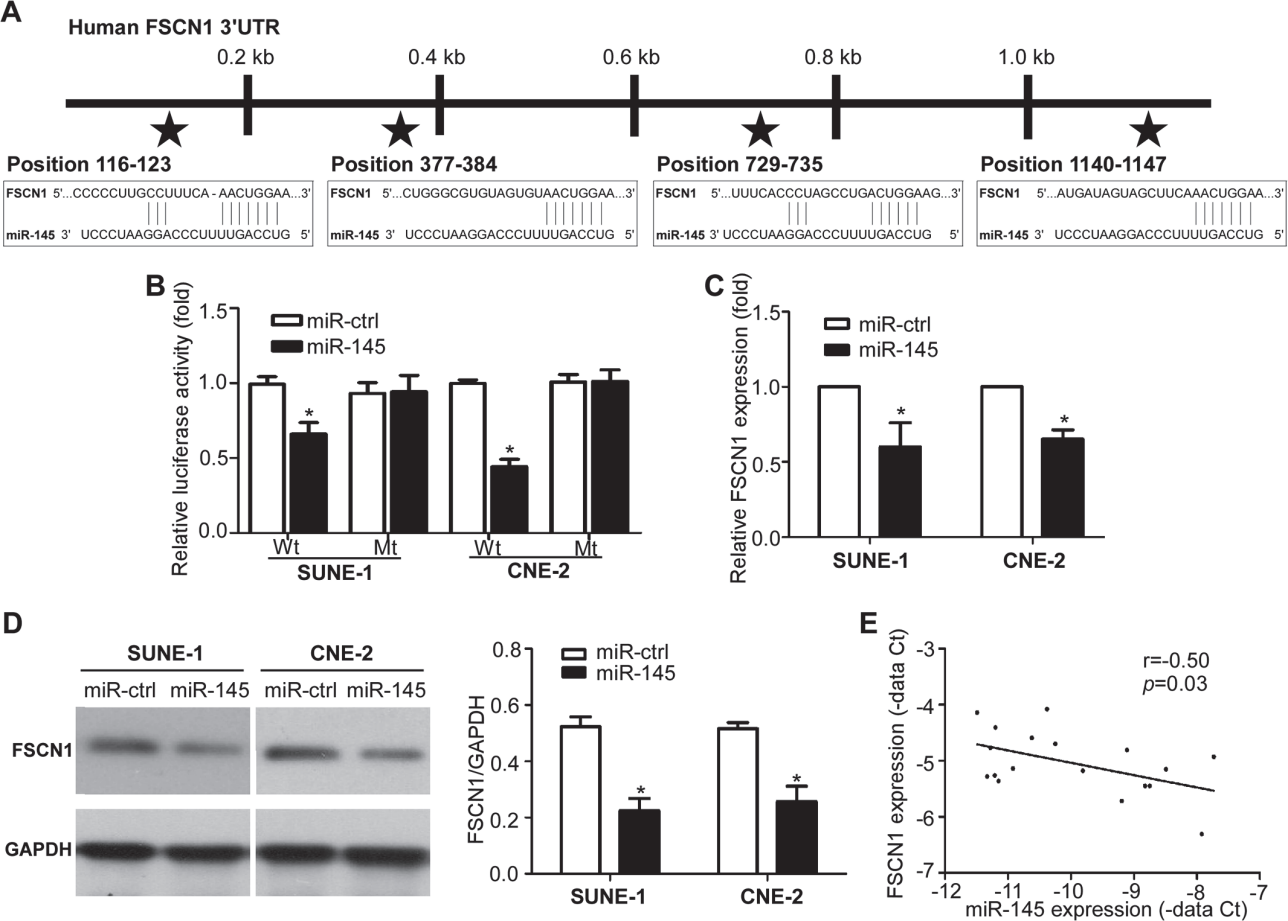
*FSCN1* is a direct target of miR-145. (A) There are four putative binding sites of miR-145 in the 3′ UTR of *FSCN1*. (B) Relative luciferase activity of SUNE-1 and CNE-2 cells after co-transfection with wild-type (Wt) or mutant (Mt) *FSCN1* 3′ UTR reporter genes and miR-145 mimics or control. (C) Quantification of *FSCN1* mRNA expression in SUNE-1 and CNE-2 cells transfected with miR-145 mimics or miRNA control. (D) Quantification of *FSCN1* protein expression in SUNE-1 and CNE-2 cells transfected with miR-145 mimics or miRNA control. (E) Spearman’s correlation analysis of miR-145 and FSCN1 expression in NPC tissues (n = 18); a significant inverse correlation was observed.

To further explore the mechanism by which miR-145 inhibit invasion and metastasis, we used three publicly databases (TargetScan, DIANA, and miRanda) to identify potential targets of miR-145, and selected *FSCN1* for further analysis. As shown in Fig 6A, there were four putative miR-145 binding sites in the 3′ UTR of *FSCN1* mRNA. We first cloned the wild-type or mutant miR-145 target sequences of the *FSCN1* 3′ UTR into luciferase reporter vectors, and did luciferase reporter gene assay. Co-transfection with miR-145 mimics obviously reduced the luciferase activity of the wild-type reporter gene, but not the mutant reporter gene (Fig 6B, *p*<0.05). Furthermore, quantitative RT-PCR and Western blotting showed that overexpressing of miR-145 remarkably reduced the mRNA and protein expression of *FSCN1* (Fig 6C and 6D, *p*<0.05). In addition, we analyzed the correlation between miR-145 and FSCN1 expression in another cohort of 18 freshly-frozen NPC samples by quantitative RT-PCR. The expression of miR-145 was inversely correlated with FSCN1 expression in the clinical NPC samples (Fig 6E, *p*<0.05). Taken together, these findings demonstrated that miR-145 could negatively regulate the expression of *FSCN1* by directly targeting the *FSCN1* 3′ UTR.

### FSCN1 is involved in NPC cell migration and invasion

**Fig 7:**
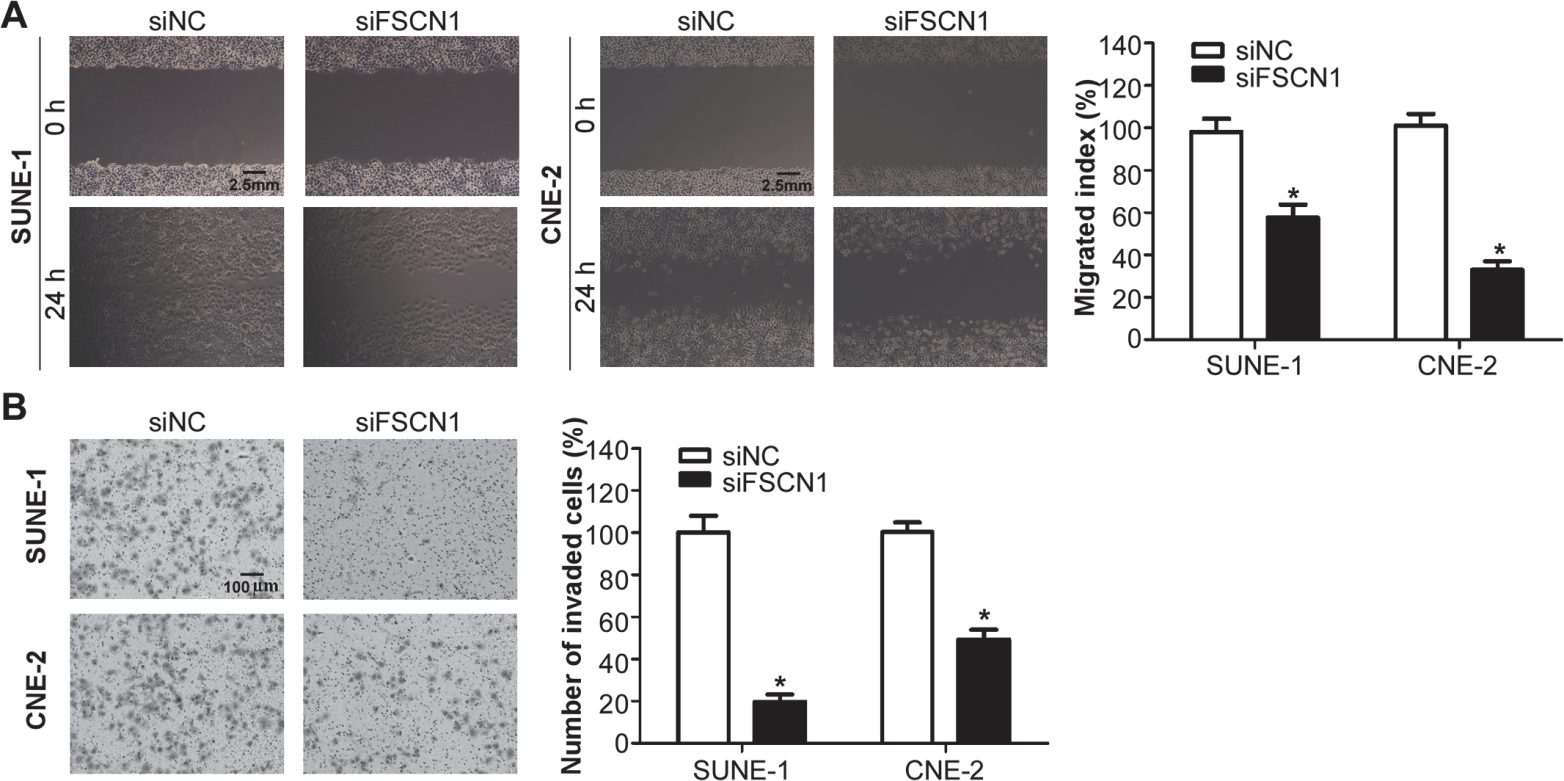
*FSCN1* is involved in NPC cell migration and invasion. (A) Representative photographs (left) and quantification (right) of the wound healing assay with SUNE-1 and CNE-2 cells transfected with siFSCN1 or siRNA control. (B) Representative photographs (left) and quantification (right) of the Transwell invasion assay with SUNE-1 and CNE-2 cells transfected with siFSCN1 or siRNA control (* *p* < 0.05; Student’s *t*-test).

Finally, to determine whether *FSCN1* could regulate migration and invasion of NPC cells, we transiently transfected SUNE-1 and CNE-2 cells with small interfering RNA siFSCN1 or siRNA control, and conducted wound healing assay and Transwell invasion assay. As shown in Fig 7A, SUNE-1 and CNE-2 cells transfected with siFSCN1 migrated slower than cells transfected with siRNA control (*p*<0.05). Moreover, the Matrigel Transwell assay showed that inhibiting *FSCN1* expression significantly suppressed the invasive ability of both NPC cell lines (Fig 7B, *p*<0.05). These observations suggested that *FSCN1* was a functional target of miR-145, which involving in NPC cell migration and invasion.

## Discussion

Based on our previous microarray analysis, we discovered that miR-145 was significantly downregulated in archived NPC tissues [13]. In our present study, we found that miR-145 was also frequently decreased in NPC cell lines and freshly frozen tissue samples. Overexpression of miR-145 inhibited NPC cell migration and invasion *in vitro*, and suppressed the formation of lung metastatic nodes *in vivo*. Furthermore, *FSCN1* was identified and verified as a direct target of miR-145, and involved in regulating NPC cell migration and invasion. Taken together, these observations demonstrated that the dysregulation of miR-145 plays important roles in the development and progression of NPC, especially in the processes of metastasis.

Metastasis is the major hallmarks of malignant tumors, and is responsible for the majority of cancer-related deaths [5]. NPC is a malignant tumor with high rates of local invasion and distant metastasis. With the considerable advances made in multimodal treatment, especially the advent of intensity-modulated radiotherapy, the local control rates for NPC has been significantly improved, and distant metastasis becomes the most common failure patterns [3–4]. Therefore, better understanding of the molecular mechanisms involving in NPC metastasis is critical for the development of novel treatments for NPC patients. MiRNAs are small noncoding double-stranded RNA molecules and serve as master regulators of gene expression at the post-transcriptionally level by base-pairing to the 3′ UTR of their target genes in a sequence-specific manner [10]. Recently, multiple studies report that miRNAs can serve as oncogenes or tumor suppressors, and are involved in regulating the processes of tumor metastasis [29–30], which may offer a novel way to explore the molecular mechanisms underlying NPC progression and to develop novel treatment strategies for NPC.

In recent years, abnormal expression of miRNAs has been reported in NPC by our lab and other research institutes [13–16]. Several studies also reported that the dysregulation of specific miRNAs was involved in NPC cell invasion and metastasis [17–21]. Recent findings have shown that miR-145 is frequently downregulated in human cancers, and functions as a tumor suppressor [22–28]. MiR-145 was reported to suppress breast cancer cell invasion and metastasis, and regulate its epithelial to mesenchymal transition [22,28]. It was also reported that miR-145 could induce colon cancer cell apoptosis, regulate cell cycle distribution, and inhibit cell growth, migration, and invasion [23–24]. Furthermore, it was found that miR-145 could inhibit cell proliferation, tumor growth, invasion, and metastasis in liver, prostate and gastric cancers [25–27]. In our recent microarray analysis, we also found that the expression of miR-145 was significantly decreased in archived NPC tissue samples [13]. However, no study has studied its biological function and mechanisms in NPC. Therefore, in our present study, we aim to further investigate the affect of miR-145 in NPC. Firstly, quantitative RT-PCR confirmed that miR-145 was obviously downregulated in NPC cell lines and freshly frozen tissues. The wound healing, Transwell migration and invasion, three-dimension spheroid invasion, and experimental lung metastasis assays demonstrated that miR-145 could significantly inhibit NPC cell migration and invasion *in vitro*, suppress the formation of lung metastatic nodes *in vivo*. Here, we firstly provide evidences that miR-145, serving as a novel anti-metastasis factor, plays important roles in preventing the progression and metastasis of NPC.

As we known, each individual miRNA has the potential to modulate multiple genes that harbor target sequence in their 3′ UTR and complement to the seed region of the miRNA [10]. Several target genes of miR-145, including mucin 1 (*MUC1*), DNA Fragmentation Factor-45 (*DFF45*), *catenin δ-1*, histone deacetylase 2 (*HDAC2*), v-ets avian erythroblastosis virus e26 oncogene homolog (*ERG*), N-cadherin (*CDH2*), and POU class 5 homeobox 1 (*OCT4*), have been verified [22–28], and on the basis of these observations, miR-145 has been acknowledged as a tumor suppressor. In our present study, we identified and verified that *FSCN1* was a direct target of miR-145 using luciferase reporter assay, which was further confirmed by the findings that ectopic expression of miR-145 could inhibit the *FSCN1* expression at both the mRNA and protein level, the expression of miR-145 was inversely correlated with FSCN1 expression in the clinical NPC samples. More interesting, *FSCN1* has been verified as the target of miR-145 in bladder cancer, esophageal squamous cell carcinoma, and prostate cancer [31–33]. Studies also reported that *FSCN1* is upregulated in malignant tumors, and is associated with the aggressive behavior of tumors by promoting cell invasiveness [34]. It has also been demonstrated that *FSCN1* was overexpressed in NPC tissues and its upregulation was correlated with poor prognosis [35]. In this report, we elucidated that silencing *FSCN1* with small interfering RNA obviously inhibited NPC cell migration and invasion. These findings demonstrate for the first time that miR-145 can inhibit NPC migration, invasion and metastasis through targeting its target gene *FSCN1*.

All together, the present study demonstrates that miR-145 functions as a tumor suppressor and has a suppressive role in the processes of NPC metastasis. We identified and verified that *FSCN1* was a direct functional target of miR-145, and involved in regulating NPC cell migration and invasion. The feature of miR-145 acting as a tumor suppressor suggests it can serve as a novel therapeutic target for cancer therapy. It would be meaningful and helpful to explore whether miR-145 may function as a therapeutic agent for patients with NPC.
